# The effects of teachers' homework follow-up practices on students' EFL performance: a randomized-group design

**DOI:** 10.3389/fpsyg.2015.01528

**Published:** 2015-10-13

**Authors:** Pedro Rosário, José C. Núñez, Guillermo Vallejo, Jennifer Cunha, Tânia Nunes, Natalia Suárez, Sonia Fuentes, Tânia Moreira

**Affiliations:** ^1^Departamento de Psicologia Aplicada, Escola de Psicologia, Universidade do MinhoBraga, Portugal; ^2^Departamento de Psicologia, Universidad de OviedoOviedo, Spain; ^3^Vicerrectoría Académica, Universidad Central de ChileSantiago de Chile, Chile; ^4^Facultad de Educación, Universidad Autónoma de ChileSantiago de Chile, Chile

**Keywords:** types of homework follow-up, academic performance, English as a Foreign Language (EFL), homework, teachers' practices

## Abstract

This study analyzed the effects of five types of homework follow-up practices (i.e., checking homework completion; answering questions about homework; checking homework orally; checking homework on the board; and collecting and grading homework) used in class by 26 teachers of English as a Foreign Language (EFL) using a randomized-group design. Once a week, for 6 weeks, the EFL teachers used a particular type of homework follow-up practice they had previously been assigned to. At the end of the 6 weeks students completed an EFL exam as an outcome measure. The results showed that three types of homework follow-up practices (i.e., checking homework orally; checking homework on the board; and collecting and grading homework) had a positive impact on students' performance, thus highlighting the role of EFL teachers in the homework process. The effect of EFL teachers' homework follow-up practices on students' performance was affected by students' prior knowledge, but not by the number of homework follow-up sessions.

## Introduction

Homework is defined as a set of school tasks assigned by teachers to be completed by students out of school (Cooper, 2001). Several studies have showed the positive impact of this instructional tool to enhance students' school performance and develop study skills, self-regulation, school engagement, discipline, and responsibility (e.g., Cooper et al., 2006; Rosário et al., 2009, 2011; Buijs and Admiraal, 2013; Hagger et al., 2015).

In the homework process teachers have two major tasks: designing and setting activities (Epstein and Van Voorhis, 2001, 2012; Trautwein et al., 2009a), and checking and/or providing homework feedback to students (Trautwein et al., 2006b; Núñez et al., 2014). Cooper (1989) called the later “classroom follow-up” (p. 87). Classroom follow-up includes feedback provided by the teacher (e.g., written comments, marking homework, and incentives; Cooper, 1989, 2001). Hattie and Timperley (2007) defined feedback as the information provided by an educational agent or the student (self) on aspects of the performance. Feedback is an important source of information for checking answers (Narciss, 2004) and improving academic performance (Nicol and Macfarlane-Dick, 2006; Shute, 2008; Duijnhouwer et al., 2012). According to Walberg and Paik (2000), feedback is “the key to maximizing the positive impact of homework” (p. 9) because teachers take advantage of the opportunity to reinforce the work that was well-done by the students or teach them something new that would help them improve their work. Moreover, Cooper (1989, 2001) argued that the way teachers manage students' homework assignments presented in classroom may influence how much students benefit from homework.

Research on homework, with a particular focus on the homework follow-up practices commonly used by teachers, has looked into various practices such as homework control perceived by students (e.g., Trautwein et al., 2006a,b), teachers' feedback on homework (Cardelle and Corno, 1981; Elawar and Corno, 1985), and feedback on homework perceived by students (e.g., Xu, 2008, 2010). Studies conducted in several countries (e.g., Germany, Hong Kong, Singapore) reported homework control (i.e., checking whether students have completed their homework) as the homework follow-up practice teachers use in class most often in elementary and middle school levels (see Trautwein et al., 2009a; Kaur, 2011; Zhu and Leung, 2012). However, studies carried out in mathematics and French as a second language concluded that controlling homework completion reported by middle school students, or controlling students' homework style reported by teachers (e.g., “By looking at a student's assignment, I can quickly tell how much effort he/she has put into it”) did not have any effect on middle school students' achievement (Trautwein et al., 2002, 2009a). To our knowledge, only the study by Trautwein et al. (2006b) found a positive predictive effect of homework control perceived by middle school students on students' homework effort in French as a Foreign Language at the student level but not at the class level.

Regarding homework feedback, Walberg and Paik (2000) described “[homework feedback as] the key to maximizing the positive impact of homework” (p. 9). In fact, the literature has evidenced a positive relationship between homework feedback and students' outcomes. For example, Xu (2008, 2010) examined the benefits of homework feedback using a measure of teacher's feedback on homework. This measure assessed middle and high school students' perceptions on topics such as: discussing homework, collecting homework, checking homework, grading homework [i.e., assigning numerical grades for homework], and counting homework completion for students' overall grade. However, Xu (2008); Xu (2010) did notanalyzed the impact of any particular feedback practice. The same author found a positive relationship between homework feedback provided by teachers (as perceived by the middle and high school students) and students' interest in homework (Xu, 2008); students' homework management (Xu, 2012; Xu and Wu, 2013); and students' homework completion (Xu, 2011). More recently, Núñez et al. (2014) analyzed the relationship between teachers' homework feedback as perceived by students from the fifth to the twelfth grade and academic achievement, and reported an indirect relationship between homework feedback and academic achievement through students' homework behaviors (e.g., amount of homework completed).

Other studies on homework have examined the effects of written feedback on students' academic outcomes. In particular, Cardelle and Corno (1981) and Elawar and Corno (1985), examined the effects of three types of written homework feedback (i.e., praise, constructive criticism, constructive criticism plus praise) using an experimental design, and concluded that student's performance when given constructive criticism plus praise was higher than when given the other two types of feedback in primary education (Elawar and Corno, 1985) or in higher education (Cardelle and Corno, 1981). These results stress how important teachers' feedback may be not only because of its positive effect on homework, but also because it provides students with information on how to improve their work (Cardelle and Corno, 1981). The synthesis by Walberg et al. (1985) confirmed the results of previous studies and showed that “commented upon or graded homework” (p.76) increased the positive effect of homework on academic achievement of elementary and secondary students.

The literature has shown the effect of some teachers' homework follow-up practices on students' homework behaviors and academic achievement (Xu, 2012; Xu and Wu, 2013; Núñez et al., 2014), yet the use of different measures and sources of information (e.g., see Trautwein et al., 2006b, 2009a) makes it difficult for researchers to draw conclusions about the benefits of the various types of homework follow-up practices. Moreover, Trautwein et al. (2006b) suggested that future studies should include other dimensions of teachers' homework practices (e.g., checking homework completion, grading homework). However, to our knowledge, research has not yet analyzed the effects of the various types of homework follow-up practices used by teachers.

To address this call, we used a quasi-experimental design in a study conducted in an authentic learning environment in order to analyze the relationship between five types of homework follow-up practices (i.e., *1, Checking homework completion*; *2, Answering questions about homework*; *3, Checking homework orally*; *4, Checking homework on the board*; *and 5, Collecting and grading homework*) used by EFL teachers and their students' performance in English. Findings may be useful to school administrators and teachers as they may learn and reflect upon the effects of the homework follow-up practices used in class, which may in turn promote homework effectiveness and school success.

Considering the scarce results of prior studies, it was not possible to establish specific hypotheses regarding the relationship between type of homework feedback and student academic performance. However, taking into account the nature of each type of feedback and its implications for student learning process, in this study we hypothesize that:

1. The types of homework feedback analyzed are differentially associated with student academic performance (increasing from types 1–5);
2. The magnitude of the impact of the types of teacher homework feedback on academic performance is associated with students' prior level of performance.

## Methods

### Participants

A randomized-group design study was conducted in which 45 EFL teachers (classes) were randomly assigned to five homework follow-up conditions (nine EFL teachers per condition). Nineteen teachers were excluded from the study for various reasons (three were laid off, six did not give an accurate report of the procedures followed or submitted the data requested, and 10 did not follow the protocol closely. In the end 26 EFL teachers (20 females) aged 28–54 participated in the study. The final distribution of the teachers per condition was as follows: Type 1 (4); Type 2 (3); Type 3 (5); Type 4 (15); Type 5 (2). Participants had 3–30 years of teaching experience (*M* = 19) and taught English to a total of 553 sixth-graders at six state schools in the north of Portugal. Students' age ranged 10–13 (*M* = 11.05; *SD* = 0.87), and there were 278 girls (50.3%) and 275 boys (49.7%).

Learning English as a foreign language is compulsory from fifth to ninth grade in all Portuguese middle schools. Middle school is divided into two stages: the first stage includes fifth and sixth grade (age range 10–11), and the second stage includes seventh to ninth grade (age range 12–14). Our study was conducted with sixth grade students, which is the last year of the first stage. English is taught in two 90-min weekly lessons. As the Portuguese public school system has not enacted any specific homework policies, teachers are free to decide on the amount, frequency, and type of homework they design. This study was carried out in accordance with the recommendations of the ethics committee of the University of Minho, with written informed consent from all subjects enrolled (i.e., teachers and their students). All subjects gave written informed consent in accordance with the Declaration of Helsinki.

### Measures

The two English performance measures used in this study were collected from the schools' secretary's office. Prior performance (used as a pretest) was obtained from students' grades in a final English exam completed at the end of the previous school year (end of June). Fifth grade EFL students from the six public schools enrolled in the study (all from the same region of the country) completed the same non-standardized exam in the end of the school year (June). This English exam comprised 30 questions on reading comprehension skills, vocabulary, and grammar which were calibrated by a group of EFL teachers from all the intervening schools.

Final academic performance (used as a posttest) was obtained from the students' grades in a final English exam set up specifically for this study and completed at the end of it (beginning of November). The posttest exam was made up of 20 questions designed to assess students' reading comprehension skills, vocabulary, grammar (contents covered in homework assignments 1, 2, 4, and 5), translation skills from English into Portuguese and vice versa, and writing of a short text (5–10 lines; contents covered in homework assignments 3 and 6). The exam lasted 45 min. Grades in the Portuguese compulsory educational system (first to ninth grade) range from 1 to 5, where 1 and 2 is fail, 3 pass, 4 good, and 5 excellent.

### Procedure

To accomplish our goal, the types of homework follow-up practices were selected from the ones identified in the literature (e.g., Walberg et al., 1985; Murphy et al., 1987; Cooper, 1989, 2001; Trautwein et al., 2006b). To learn which homework follow-up practices were used by teachers in class to deal with students' delivery of homework assignments, 15 Portuguese middle school EFL teachers were invited to participate in two focus group interviews (one group comprised seven teachers and the other eight teachers). Note that these EFL teachers did not participate in the research intervention.

Findings from this ancillary study allowed the confirmation of the two homework follow-up practices reported in the literature (i.e., *checking homework completion, collecting, and grading homework*), and identified three additional practices which were used in the current study. Data from this ancillary study will not be described in detail due to space constraints. Nevertheless, some examples of each homework practice are presented in Table 1.

**Table 1:** **The five types of homework follow-up practices exemplified with quotations from the participating teachers in the focus group interviews**.

Type 1	*Checking homework completion*:
	“[in class] I just check and note down whether students did their homework. This is the only type of homework feedback I can provide…I wish had time for more” (F2P7)
Type 2	*Answering questions about homework:*
	“[in class] I just ask students if they did or did not understand their homework tasks. If any, I just answer questions about homework because I want to start the class as soon as possible. You know, I need to teach them all the contents in the course program and…” (F1P1)
Type 3	*Checking homework orally*:
	“I usually check homework orally. By answering questions about homework tasks I have the opportunity to explain and suggest strategies to improve learning” (F2P8)
Type 4	*Checking homework on the board:*
	“I always check homework on the board because I want to see if students understood the contents and my explanations,” (F1P3)
Type 5	*Collecting and grading homework:*
	“I collect students' notebooks […] because I learned that my students do better when I comment upon and grade their homework assignments…” (F1P6)


Five homework follow-up practices were included in our study as follows: (1) Checking homework completion; (2) Answering questions about homework; (3) Checking homework orally; (4) Checking homework on the board; and (5) Collecting and grading homework. Types 1 and 5 were based on the literature (Walberg et al., 1985; Murphy et al., 1987; Trautwein et al., 2006b), and types 2–4 emerged in the focus group interviews with the EFL teachers, and were included in this study because of their local relevance.

Data were collected at the beginning of the school year (between mid-September and end of October) after obtaining permission from schools' head offices. EFL teachers confirmed their intention to participate via email, and from those who had confirmed participation, 45 and their students were randomly selected. Two weeks before the beginning of the study, the 45 EFL teachers participated in a 4-h information meeting which explained the project's aims and the research design in detail (e.g., analysis and discussion of the format and content of the English exam to assess students' performance; and information on the frequency, number, and type of homework assignments; guidelines to mark the homework assignments; and the five types of homework follow-up practices). Additionally, teachers were informed that they would be randomly assigned to an experimental condition, and the associated methodological reasons were discussed with the participants. All teachers agreed and were then randomly assigned to one of the five homework follow-up conditions (nine teachers per condition). However, only 26 teachers completed the study (see Section Participants). At the meeting, all teachers agreed to assign homework to their students only once a week (in the first class of the week) and to check homework completion in the following class using the type of homework follow-up condition they had been assigned to. The six homework assignments were extracted from the English textbook and common to all participants. Two different types of homework were assigned. The first type had reading comprehension, vocabulary, and grammar questions (homework assignments 1, 2, 4, and 5). The second type (homework assignments 3 and 6) had a translation exercise from English into Portuguese and vice versa, and writing of a short text in English (5–10 lines). After selecting the homework exercises, teachers worked on the guidelines to mark each homework assignment, and built a grade tracking sheet to be filled in with information regarding each student and each homework. The grade tracking sheet filled in with students data was delivered to researchers in the following class.

At the end of each lesson, the students noted down the instructions for the homework assignment in their notebooks and completed it out of class.

The researchers gave the EFL teachers extensive training on the homework follow-up practices in order to guarantee that all the participants under each condition followed the same protocol. During the information meeting a combination of theory and practice, open discussion, and role-playing exercises were used.

For each condition, the protocol was as follows. For homework follow-up condition no. 1 (*checking homework completion*), the teacher began the class asking students whether they had completed their homework assignment (i.e., yes, no) and recorded the data on a homework assignment sheet. For homework follow-up condition no. 2 (*answering questions about homework*), the teacher began the class asking students if they had any questions about the homework assignment (e.g., Please, ask any questions if there is something in the homework which you did not understand.), in which case the teacher would answer them. For homework follow-up condition no. 3 (*checking homework orally*), the teacher began the class checking homework orally. Under this condition the teachers proactively read the homework previously assigned to students and orally checked all the tasks or questions (i.e., the teacher read the questions and students answered them aloud, followed by an explanation of the mistakes made by students). For homework follow-up condition no. 4 (*checking homework on the board*), the teacher started the class by writing the answer to each of the homework questions on the board. Following the explanation to a specific question or task, the EFL teachers explicitly asked the class: “Do you have any other questions?” and moved on to the next question. In the case of homework follow-up condition no. 5 (*collecting and grading homework*), the teacher began the class handing out individually checked and graded homework to students. For homework assignments 1, 2, 4, and 5 (i.e., reading comprehension, vocabulary, and grammar questions) the EFL teachers pointed out which of answers were incorrect, and provided the correct answer. A numerical grade for each of the exercises and a global grade were awarded. For the second type of homework assignments (3 and 6; i.e., translation from English into Portuguese and vice versa, and writing of a short text in English), the EFL teachers made comments on the text in terms of contents and style, and gave a numerical grade. Students were encouraged to read the teachers' comments on their homework and asked if they had any questions.

To guarantee the reliability of the measurements (i.e., whether the EFL teachers followed the protocol), three research assistants were present at the beginning of each class. For 15 min, the research assistants took notes on the type of homework follow-up used by the teachers using a diary log. The level of overall agreement among the research assistants was estimated with Fleiss's Kappa (Fleiss, 1981). According to Landis and Koch (1977), the reliability among the research assistants may be rated as good (κ = 0.746; *p* < 0.001).

Data from the 19 EFL teachers who did not follow the protocol for their assigned homework follow-up condition were not included in the data set. Three weeks after the study, EFL teachers attended a 2-h post-research evaluation meeting with the aim to discuss their experience (e.g., comments and suggestions that could help in future studies; difficulties faced in implementing their experimental condition; reasons for not following the protocol), and analyze preliminary data. At the end of the six homework follow-up sessions, students completed a final English exam as a measure of academic performance (posttest).

### Data analysis

Each of the five homework follow-up practices was to be administered by the same number of EFL teachers (nine). However, as mentioned above, 19 EFL teachers were excluded from the study, which led to an uneven distribution of the participating teachers under the five conditions. As the number of homework follow-up sessions was not even in terms of type, it was not possible to guarantee the independence of these two variables (i.e., number of homework follow-up and type of homework follow-up practice). Thus, the amount of treatments (number of homework follow-up sessions) was taken as a control variable. The effect of the EFL teachers nested within the treatment levels (the five homework follow-up practices) was also controlled, but within the type of design (cluster randomized design). Furthermore, students' prior performance was controlled because of its potential to influence the relationship between homework and academic achievement (Trautwein et al., 2002, 2009b).

Finally, the design included an independent variable (type of homework follow-up), a dependent variable (post-homework follow-up academic performance), and two covariates (number of homework follow-up sessions administered and performance prior to homework follow-up). The statistical treatment of the data was carried out using analysis of covariance (ANCOVA). Data analysis followed a two-stage strategy. First, we examined whether prior performance (pretest) significantly explained academic performance at posttest (which led to testing whether the regression slopes were null). If the result was positive, it would not be necessary to include any covariate in the model, and an ANOVA model would be fitted. On the other hand, if the result was negative, second stage, it would be necessary to verify whether the regression slopes were parallel (that is, whether the relationship between prior and final performance was similar across the different types of homework follow-up). Finally, in case the parallelism assumption were accepted, paired comparisons between the adjusted homework follow-up type variable measures (i.e., purged of covariate correlations) would be run using the method based on the false discovery rate (FDR) developed by Benjamini and Hochberg (1995) (BH).

Data were analyzed using SAS version 9.4 [SAS Institute, Inc., (SAS), 2013]. The hypotheses referring to nullity and parallelism of the regression slopes were tested using SAS PROC MIXED with the solution proposed by Kenward and Roger (2009). PROC MIXED allows the use of a linear model that relaxes the assumption of constant variance (for details, see Vallejo et al., 2010; Vallejo and Ato, 2012). The *post-hoc* contrasts were done using the ESTIMATE expression in SAS PROC MIXED and the BH/FDR option in SAS PROC MULTITEST.

## Results

### Descriptive statistics

Table 2 shows the descriptive statistics of the homework follow-up type variable and the two covariates (prior performance and the number of homework follow-up sessions).

**Table 2:** **Descriptive statistics of the variable homework follow-up practice and covariates (prior performance and number of times feedback is provided)**.

		***N***	**Min**.	**Max**.	***M***	***SD***
Prior performance		553	2	5	3.55	0.92
Final performance		553	2	5	3.57	0.97
Number of sessions		6	1	6	4.43	01.62
Homework follow-up		5	1	5	3.18	1.20
Homework follow-up _1	Pretest	85	2	5	3.36	0.88
	Posttest	85	1	5	3.27	0.99
Homework follow-up _2	Pretest	65	2	5	3.34	0.87
	Posttest	65	2	5	3.26	0.94
Homework follow-up _3	Pretest	104	2	5	3.42	0.93
	Posttest	104	2	5	3.52	0.97
Homework follow-up _4	Pretest	264	2	5	3.68	0.96
	Posttest	264	2	5	3.73	0.97
Homework follow-up _5	Pretest	35	2	5	3.74	0.78
	Posttest	35	2	5	3.83	0.78

*N, total number of subjects; Min, minimum value; Max, maximum value; SD, standard deviation; M, mean; homework follow-up _1, checking homework completion; homework follow-up _2, answering questions about homework; homework follow-up _3, checking homework orally; homework follow-up _4, checking homework on the board; homework follow-up _5, collecting and grading homework; Pretest, performance before homework follow-up; posttest, performance after homework follow-up*.

### Analysis of covariance

#### Null regression curve test

To determine whether prior performance (pretest) significantly explained academic performance at posttest, a type III sum of squares model without an intercept was created. This model included the homework follow-up type (A), and interactions of homework follow-up type with the covariates prior performance (*X*_1_), and number of homework follow-up sessions (*X*_2_); that is, *A* × *X*_1_ and *A* × *X*_2_. The information obtained in this analysis allowed to consider regression slopes for each level of the homework follow-up type variable, and to evaluate its nullity and, to a certain extent, its parallelism. In summary, the technique used aimed to determine whether covariates (number of homework follow-up sessions administered and performance prior to homework follow-up) modified the interaction between homework follow-up type and final performance. Table 3 addresses this question and shows two model effects: the principal effect (A) and secondary effects (*A* × *X*_1_ and *A* × *X*_2_).

**Table 3:** **Estimators of interaction parameters obtained in the first modeling stage after creating a regression model without an intercept**.

**Effect**	**Estimate**	***SE***	***DF***	***T*-value**	***Pr* > |*t*|**
[*A* = 1.00]	−0.04	0.34	538	−0.11	0.915
[*A* = 2.00]	−0.39	0.42	538	−0.92	0.360
[*A* = 3.00]	0.64	0.24	538	2.67	0.008
[*A* = 4.00]	0.71	0.24	538	2.96	0.003
[*A* = 5.00]	0.41	0.27	538	1.53	0.127
[*A* = 1.00] × Prior performance	0.96	0.10	538	10.56	< 0.001
[*A* = 2.00] × Prior performance	0.96	0.09	538	10.47	< 0.001
[*A* = 3.00] × Prior performance	0.87	0.06	538	15.37	< 0.001
[*A* = 4.00] × Prior performance	0.86	0.033	538	26.37	< 0.001
[*A* = 5.00] × Prior performance	0.94	0.063	538	14.92	< 0.001
[*A* = 1.00] × Number of sessions	0.02	0.03	538	0.59	0.552
[*A* = 2.00] × Number of sessions	0.15	0.06	538	2.56	0.011
[*A* = 3.00] × Number of sessions	−0.03	0.04	538	−0.76	0.446
[*A* = 4.00] × Number of sessions	−0.03	0.04	538	−0.85	0.398
[*A* = 5.00] × Number of sessions	−0.04	0.04	538	−0.85	0.395

*[A = 1,…,5], homework follow-up practices*.

Data show that all regression coefficients involving the prior performance covariate were statistically significant (*p* < 0.001) with very similar levels for the homework follow-up type variable (between *p* = 0.86 and 0.96). Thus, we may conclude that the slopes were not null. A strong similarity was also observed between the regression coefficients, which indicates that the number of homework follow-up sessions, with the exception of the coefficient corresponding to level 2 of the homework follow-up type variable (*b*_*A*2 × *S*_ = 0.15), was also statistically significant (*p* = 0.011).

#### Parallel regression slope test

To test the hypothesis of regression slope parallelism for the covariates prior performance (*X*_1_) and number of homework follow-up sessions (*X*_2_) on final academic performance, the interaction components *A* × *X*_1_ and *A* × *X*_2_ of Model A shown in Table 4 are particularly interesting.

**Table 4:** **Results of fitting three ANCOVA models and one ANOVA model during the second stage of the modeling strategy**.

**Fixed effects**	**Model A**			**Model B**			**Model C**			**ANOVA model**		
	**DF**	***F*-value**	***Pr* > *F***	**DF**	***F*-value**	***Pr* > *F***	**DF**	***F*-value**	***Pr* > *F***	**DF**	***F*-value**	***Pr* > *F***
	**Num Den**			**Num Den**			**Num Den**			**Num Den**		
*A*	4, 162	1.92	0.109	4, 183	2.81	0.027	4, 159	2.85	0.027	4, 150	6.99	< 0.001
*X*_1_	1, 242	846.74	< 0.001	1, 465	1338.89	< 0.001	1, 467	1345.16	< 0.001			
*X*_2_	1, 252	0.54	0.464	1, 373	0.16	0.689						
*A* × *X*_1_	4, 160	0.62	0.646									
*A* × *X*_2_	4, 144	2.20	0.071									
**Cov Parm**	**Estimate**	***Z*****-Value**	***Pr*** > ***Z***	**Estimate**	***Z*****-Value**	***Pr*** > ***Z***	**Estimate**	***Z*****-Value**	***Pr*** > ***Z***	**Estimate**	***Z*****-Value**	***Pr*** > ***Z***
UN (1)	0.43	6.41	< 0.001	0.42	6.46	< 0.001	0.42	6.48	< 0.001	0.98	6.52	< 0.001
UN (2)	0.31	5.57	< 0.001	0.34	5.60	< 0.001	0.34	5.66	< 0.001	0.88	5.66	< 0.001
UN (3)	0.28	7.14	< 0.001	0.28	7.16	< 0.001	0.28	7.17	< 0.001	0.94	7.14	< 0.001
UN (4)	0.26	11.42	< 0.001	0.26	11.45	< 0.001	0.26	11.46	< 0.001	0.94	11.47	< 0.001
UN (5)	0.08	4.01	< 0.001	0.08	4.09	< 0.001	0.09	4.11	< 0.001	0.62	4.12	< 0.001
T/A	0.00	0.15	0.44									
Fit Statist	AIC	BIC		AIC	BIC		AIC	BIC		AIC	BIC	
Value	900.1	921.9		889.8	911.3		875.0	896.6		1539.5	1549.9	

*A, homework follow-up type; X_1_, previous grade; X_2_, number of homework follow-up sessions; UN (1, 2, 3, 4, 5), variance of each homework follow-up type; T/A, teachers nested within the homework follow-up type variable; DF_Num_, degrees of freedom numerator; DF_Den_, degrees of freedom denominator*.

The data show that the regression slope parallelism hypothesis was not rejected [*F*_(4, 160)_ = 0.62, *p* = 0.646 and *F*_(4, 144)_ = 2.20, *p* = 0.071], although the interaction between the number of homework follow-up sessions and the type of homework follow-up turned out to be marginally non-significant. Thus, we provisionally adopted the ANCOVA model that used equal slopes to describe the influence of the covariates on homework follow-up type. Note that the variance component of the students who received homework follow-up type no. 1 was approximately five times the variance of the students receiving type no. 5. Thus, to control the heterogeneity of the data, the GROUP expression in SAS PROC MIXED was used with the solution proposed by Kenward–Roger to adjust for the degrees of freedom (Kenward and Roger, 2009). Moreover, the variance component referring to EFL teachers nested within the homework follow-up types was not statistically significant (*z* = 0.15, *p* = 0.44), so we proceeded with the single-level ANCOVA model.

Findings indicate that the differences among the various homework follow-up types do not depend on the teacher that uses them. This preliminary result stresses the relevance of conducting multilevel designs analyzing data at two levels, students and class. This finding is aligned with those of Rosário et al. (2013) which found a small effect in the relationship between teachers' reported approaches to teaching and students' reported approaches to learning.

Table 4 also shows information regarding the fit of other ANCOVA models with identical slopes: Model B and Model C. Model B shows that the types of homework follow-up did not differ in terms of the number of homework follow-up sessions provided by the EFL teachers (*X*_2_), [*F*_(1, 373)_ = 0.16, *p* = 0.689]. Note that the ANCOVA model with equal regression slope that left out the number of homework follow-up sessions (Model C) was more parsimonious and showed the best fit. The model with the fewest information criteria, Akaike information criteria (AIC) and Bayesian information criteria (BIC), is the model that best fits the data.

**Figure 1:**
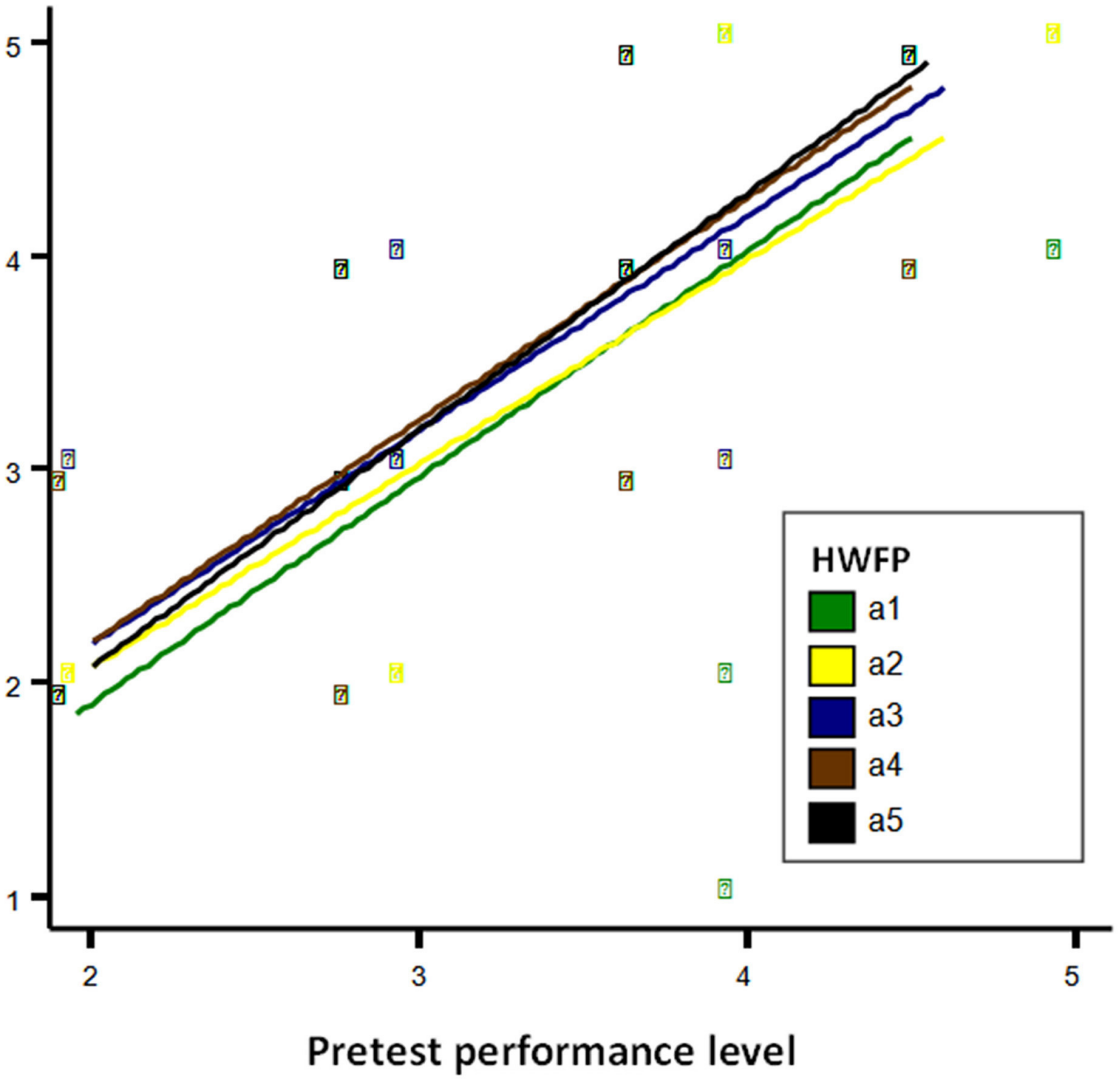
**Pretest performance level**.

The ANCOVA model with equal slopes is shown in Figure 1. The essential characteristic of the model is worth noting: separate regression lines for each type of homework follow-up and approximately parallel slopes among the homework follow-up types. Figure 1 also shows two subsets of means, each with means that barely differed from each other and were thus considered equal from a statistical standpoint. These subsets encompassed, on the one hand, the first two levels of the homework follow-up type variable (types 1 and 2), and on the other hand, the three last levels of the variable. The equal regression slope (*b* = 0.882) between prior performance and final performance, averaging all levels of homework follow-up type, was statistically significant [*t*_(467)_ = 36.86, *p* < 0.001].

#### Comparisons between the adjusted homework follow-up type means

The common slope (*b* = 0.882) was used to calculate the final performance means adjusted to the effect of the prior performance covariate. Purged of the correlation with the prior performance covariate, the adjusted final EFL performance means were *A*_1_ = 3.14; *A*_2_ = 3.11; *A*_3_ = 3.44; *A*_4_ = 3.88; and *A*_5_ = 4.03.

**Figure 2:**
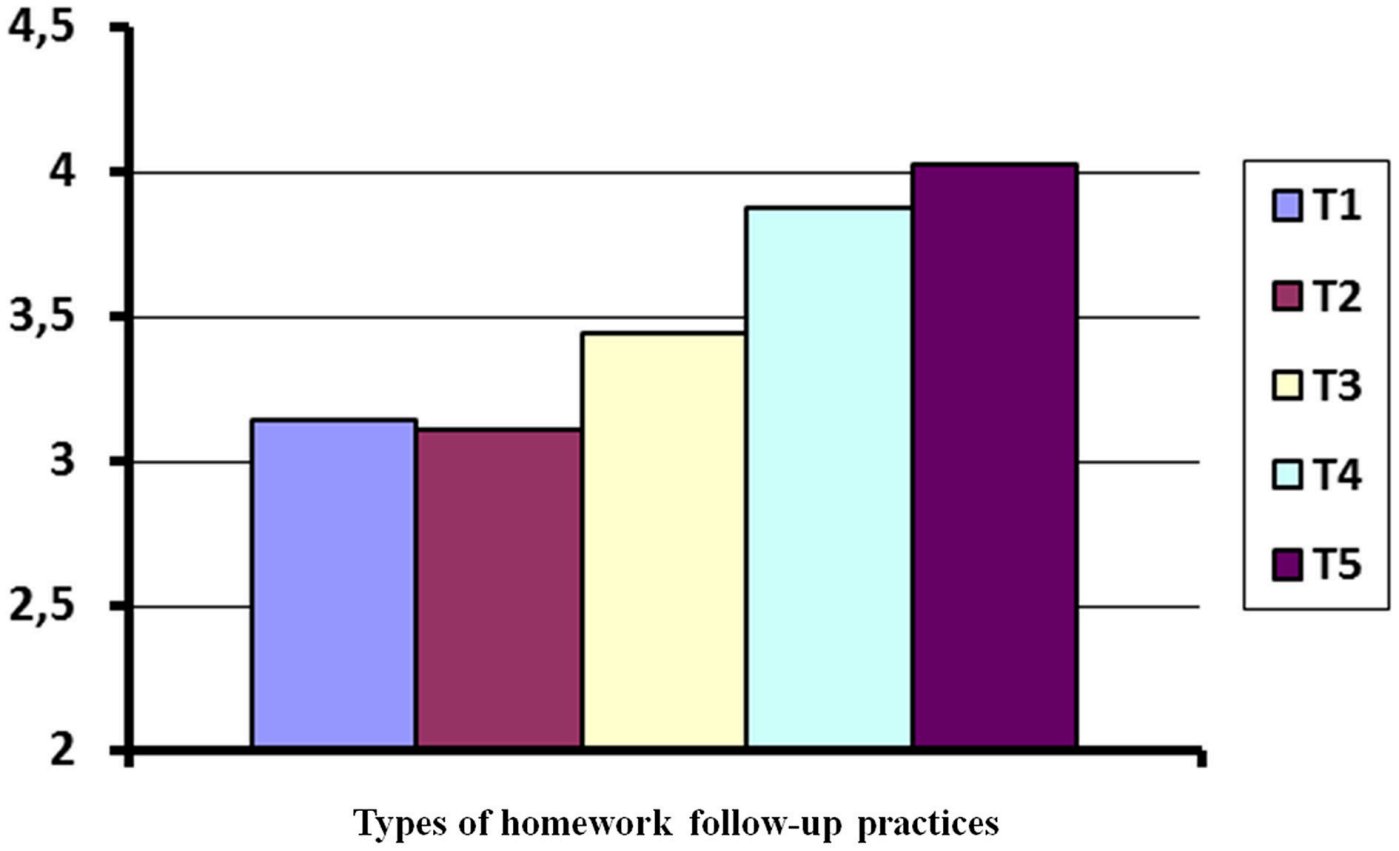
**Types of homework follow-up practices**.

Given the two homogeneous subsets of means previously detected, the family of pairwise comparisons that appear in Table 5 was tested. To control for the probability of making one or more type I errors at the chosen level of significance (α = 0.05) for the specified family or group of contrasts, assuming heterogeneity, the ESTIMATE expression in SAS PROC MIXED was used, as was the BH/FDR option in SAS PROC MULTITEST. As indicated in the last column of Table 5, the procedure detected statistically significant differences (*p* < 0.05) in five of the six contrasts analyzed (see Figure 2 as well).

**Table 5:** **Pairwise comparisons between the homework follow-up practices based on ANCOVA BH/FDR that controlled for prior performance**.

**Levels**	**Estimate**	***SE***	***DF***	***T*-value**	***P***	**RAW_P**	**fdr_p**
A1-A3	−0.19	0.09	161	−2.14	< 0.03	0.034	0.050
A1-A4	−0.18	0.08	120	−2.29	< 0.02	0.02	0.050
A1-A5	−0.22	0.09	119	−2.61	< 0.01	0.01	0.050
A2-A3	−0.17	0.09	126	−1.95	< 0.05	0.05	0.053
A2-A4	−0.16	0.08	91	−2.07	< 0.04	0.04	0.050
A2-A5	−0.21	0.09	99	−2.41	< 0.02	0.02	0.050

*[A = 1,…,5], homework follow-up practices*.

## Discussion of results

This study analyzed whether the relationship between academic performance and homework follow-up practices depended on the type of homework follow-up practice used in class. We found that the five types of homework feedback were associated with student academic performance, despite the unbalanced number of teachers in each condition, and the low number of sessions (six sessions). The magnitude of the effects found was small, which may be due to the two previously mentioned limitations. Data from the ancillary analysis collected in the two focus groups run to identify the types of homework follow-up used by EFL teachers in class, and data from the post-research evaluation meeting run with the participating teachers contributed to the discussion of our findings.

### Types of EFL teachers' homework follow-up practices and academic performance

As Model C (see Table 4) shows, and once the effect of the pretest was controlled for, the differences among the types of EFL teachers' homework follow-up practices on students' performance were statistically significant, as hypothesized. Moreover, considering the positive value of the coefficients shown in Table 4, the data indicate that students' performance improved from homework follow-up types 1–5 (see also Figure 2), and also that the differences between the five homework follow-up types are not of the same magnitude. In fact, after checking the error rate for comparison family using the FDR procedure, two homogeneous subsets of treatment means were identified. The first subset encompassed homework follow-up types 1 and 2, whereas the second accounted for homework follow-up types 3–5. As shown in Table 5, significant differences were found between adjusted treatments' means for both subsets (homework follow-up types 1 and 2 vs. homework follow-up types 3–5).

What are the commonalities and differences between these two subsets of homework follow-up types that could help explain findings? Homework follow-up types 1 and 2 did not yield differences in school performance. One possible explanation might be that neither of these types of homework follow-up provides specific information about the mistakes made by students; information which could help them improve their learning in a similar way to when EFL teachers provide feedback (Hattie and Timperley, 2007). Besides, as the control for homework completion is low for these two types of homework follow-up practices, students may not have put the appropriate effort to complete the homework. The following statement was shared by most of the teachers that participated in the focus group and may help explain this latter finding: “[in class] I only ask students if they have done their homework. I know that this strategy does not help them correct their mistakes, but if I don't do it, I suspect they will give up doing their homework …” (F2P3).

In homework follow-up type 2, EFL teachers only addressed difficulties mentioned by the students, so some mistakes may have not been addressed and checked by the EFL teachers. This type of practice does not provide feedback to students. As the following quotation from a participant in the focus group revealed: “At the beginning of the class, I specifically ask students if they have any questions about their homework. The truth is, students who struggle to learn seldom ask questions…I guess that they don't do their homework, or they copy the answers from peers during the break, and just asking questions does not help a lot…but they are 28 in class.” (F2P4).

The second group of homework follow-up practices includes types 3–5. Our data indicate that there were no statistically significant differences among these three types of homework follow-up (intra-group comparisons) at posttest performance (see Table 4). Under each of these three conditions (homework follow-up types 3–5) homework contents were checked by the teacher. In these three types of homework follow-up, students experienced opportunities to analyze EFL teachers' explanations and to check their mistakes, which may help explain our findings and those of previous studies (see Cardelle and Corno, 1981; Elawar and Corno, 1985).

According to Cooper's model (1989, 2001), homework follow-up type 5 may be considered the homework feedback practice, because when EFL teachers grade students' assignments and provide individual feedback, students' learning improve. This idea was mentioned by one of our participants: “I collect students' exercise books, not every day, but often enough. That is because I've learned that my students improve whenever I comment upon and grade their homework assignments. I wish I had time to do this regularly…That would be real feedback, that's for sure.” (F1P6).

When analyzing students' conceptions of feedback, Peterson and Irving (2008) concluded that students believe that having their reports graded is a “clearer and more honest” (p. 246) type of feedback. These authors also argued that good grades generate a tangible evidence of students' work for parents, which may also give way to another opportunity for feedback(e.g., praise) delivered by parents and peers (Núñez et al., 2015). It is likely that students see graded homework more worthwhile when compared to other types of homework follow-up practices (e.g., answering questions about homework). This idea supports studies which found a positive association between homework effort and achievement (e.g., Trautwein et al., 2006b, 2009b). Walberg et al. (1985) claimed that graded homework has a powerful effect on learning. However, Trautwein et al. (2009a) alerted that graded homework may have a negative impact whenever experienced as overcontrolling, as “…students may feel tempted to copy from high-achieving classmates to escape negative consequences” (p. 185). These findings (Trautwein et al., 2006b, 2009a,b), aligned with ours, suggest the need to analyze homework feedback in more depth. For example, there are several variables that were not considered in the current research (e.g., number of students per class, number of different grade levels teachers are teaching or number of different classes teachers teach, different level of students' expertise in class, type of content domain; but also career related issues such as frozen salaries, reduced retirement costs), which may help explain our results.

We also noticed that the effect of EFL teachers' homework follow-up practices on performance was affected by students' prior performance, confirming our second hypothesis, but not by the number of homework follow-up sessions (i.e., the number of homework follow-up sessions was only marginally non-significant as a secondary factor, not as the principal factor). A quotation from a teacher under the third condition may help illustrate this finding: “reflecting on my experience under condition 3 [checking homework orally], I can tell that students' prior knowledge was very important for explaining the variations in the efficacy of this strategy. Some of my students, for example, attend language schools and master vocabulary and grammar, but others clearly need extra help. For example, checking homework on the board so that students may copy the answers and study them at home would be very beneficial for many of my students” (M15).

The results of this preliminary study were obtained in a real learning environment and focused on homework follow-up practices commonly used by EFL teachers. We acknowledge the difficulties to set up and run a randomized-group design in a real learning environment (i.e., motivating teachers to participate, training teachers to follow the protocol, control the process). Still, we believe in the importance of collecting data on-task. Plus, we consider that our preliminary findings may help teachers and school administrators to organize school-based teachers' training and educational policies on homework. For example, studies conducted in several countries (e.g., Germany, Hong Kong, Singapore, Israel) reported that checking homework completion is the homework follow-up practice most often used by teachers to keep track of students' homework (e.g., Trautwein et al., 2009a; Kaur, 2011; Zhu and Leung, 2012), and in some cases the only homework follow-up practice used in class (e.g., see Kukliansky et al., 2014). However, this type of homework follow-up does not provide students with appropriate information on how they may improve their learning. Our data show that, when EFL teachers offer individual and specific information to help student progress (e.g., homework correction, graded homework), the impact on school performance is higher, even when this help is provided for only 6 weeks. This main finding, that should be further investigated, may help teachers' in class practices and contribute to foster students' behaviors toward homework and school achievement.

In sum, our findings indicate that the time and effort teachers devote assessing, presenting, and discussing homework with students is worth the effort. In fact, students consider limited feedback an impediment to homework completion, and recognize teacher's feedback as a homework completion facilitator (Bang, 2011).

During the focus group interviews, and consistent with findings by Rosário et al. (2015), several EFL teachers stressed that, despite their positive belief about the efficacy of delivering feedback to students, they do not find the necessary time to provide feedback in class (e.g., comment on homework and grading homework). This is due to, among other reasons, the long list of contents to cover in class and the large number of students per class. Pelletier et al.'s (2002) show that the major constraint perceived by teachers in their job is related to the pressure to follow the school curriculum. Data from the focus group helped understand our findings, and highlights the need for school administrators to become aware of the educational constraints faced daily by EFL teachers at school and to find alternatives to support the use of in class homework follow-up practices. Thus, we believe that teachers, directly, and students, indirectly, would benefit from teacher training on effective homework follow-up practices with a focus on, for example, how to manage the extensive curriculum and time, and learning about different homework follow-up practices, mainly feedback. Some authors (e.g., Elawar and Corno, 1985; Epstein and Van Voorhis, 2012; Núñez et al., 2014; Rosário et al., 2015) have warned about the importance of organizing school-based teacher training with an emphasis on homework (i.e., purposes of homework, homework feedback type, amount of homework assigned, schools homework policies, and written homework feedback practices). With the focus group interviews we learned that several EFL teachers did not differentiate feedback from other homework follow-up practices, such as checking homework completion (e.g., see F2P7 statement, Table 1). EFL teachers termed all the homework follow-up practices used in class as feedback, despite the fact that some of these practices did not deliver useful information to improve the quality of students' homework and promote progress. These data suggest a need to foster opportunities for teachers to reflect upon their in-class instructional practices (e.g., type and purposes of the homework assigned, number and type of questions asked in class) and its impact on the quality of the learning process. For example, school-based teacher training focusing on discussing the various types of homework follow-up practices and their impact on homework quality and academic achievement would enhance teachers' practice and contribute to improve their approaches to teaching (Rosário et al., 2013).

### Limitations of the study and future research

This study is a preliminary examination of the relationship between five types of EFL teachers' homework follow-up practices and performance in the EFL class. Therefore, some limitations must be addressed as they may play a role in our findings. First, participating EFL teachers were assigned to one and only one of five homework follow-up conditions, but 19 of them were excluded for not adhering to the protocol. As a result, the number of EFL teachers under each condition was unbalanced, especially in the case of homework follow-up condition number 5. This fact should be considered when analyzing conclusions.

Several reasons may explain why 19 EFL teachers were excluded from our research protocol (i.e., three were laid off, six did not report the work done correctly or submitted the data requested, and ten did not followed the protocol closely). Nevertheless, during the post-research evaluation meeting the EFL teachers addressed this topic which helped understand their motives for not adhering to the protocol. For example: “I'm sorry for abandoning your research, but I couldn't collect and grade homework every week. I have 30 students in class, as you know, and it was impossible for me to spend so many hours grading.” (M7). Our findings suggest that teachers' attitudes toward homework follow-up practices are important, as well as the need to set educational environments that may facilitate their use in class.

We acknowledged the difficulty of carrying out experimental studies in authentic teaching and learning environments. Nevertheless, we decided to address the call by Trautwein et al. (2006b), and investigate teachers' homework practices as ecologically valid as possible in the natural learning environment of teachers and students.

Future studies should find a way to combine an optimal variable control model and an authentic learning environment.

Second, a mixed type of homework follow-up practices (e.g., combining homework control and checking homework on the board) was not considered in the current study as an additional level of the independent variable. In fact, some of the excluded EFL teachers highlighted the benefits of combining various homework follow-up practices, as one EFL teacher remarked: “I was “assigned” condition 5 [collecting and grading homework], but grading and noting homework every week is too demanding, as I have five more sixth grade classes to teach. So, although I am certain that giving individualized feedback is better for my students, I couldn't do it for the six homework assignments as required. In some sessions I checked homework orally.” (M24). Thus, future studies should consider the possibility of analyzing the impact of different combinations of types of homework follow-up practices. Our research focused on sixth grade EFL teachers only. To our knowledge, there are no studies examining the impact of homework follow-up practices in different education levels, but it is plausible that the type and intensity of the homework-follow up practices used by teachers may vary from one educational level to another. Hence, it would be interesting to examine whether our findings may be replicated in other grade levels, or in different subjects. Furthermore, it would be beneficial to conduct this study in other countries in order to explore whether the follow-up practices identified by EFL Portuguese teachers match those found in other teaching and learning cultures.

Third, the fact that in our study the differences found were small suggests the importance of examining the type of homework follow-up used and students' interpretation of teachers' practice. Future studies may analyze the hypothesis that students' behavior toward teacher homework follow-up practices (e.g., how students perceive their teachers' homework follow-up practices; what students do with the homework feedback information given by teachers) mediates the effect of homework on student learning and performance. In fact, the way students benefit from their teachers' homework follow-up practice may help explain the impact of these practices on students' homework performance and academic achievement. Future studies may also consider conducting more large-scale studies (i.e., with optimal sample sizes) using multilevel designs aimed at analyzing how student variables (e.g., cognitive, motivational, and affective) mediate the relationship between teacher homework follow-up type and students' learning and academic performance.

Finally, future research could also consider conducting qualitative research to analyze teachers' conceptions of homework follow-up practices, mainly feedback (Cunha et al., 2015). This information may be very useful to improving homework feedback measures in future quantitative studies. Investigating teachers' conceptions of homework follow-up practices may help identify other homework feedback practices implemented in authentic learning environments. It may also help understand the reasons why teachers use specific types of homework feedback, and explore the constraints daily faced in class when giving homework feedback. As one teacher in the focus group claimed: “Unfortunately, I don't have time to collect and grade homework, because I have too many students and the content that I have to cover each term is vast. So I just check whether all students completed their homework” (F2P1).

## Funding

This research was supported by the Portuguese Foundation for Science and Technology and the Portuguese Ministry of Education and Science through national funds and when applicable co-financed by FEDER under the PT2020 Partnership Agreement (UID/PSI/01662/2013), by the Spanish Ministry of Education and Science (Proyects: EDU2014-57571-P and PSI-2011-23395) and by Council of Economy and Employment of the Government of the Principality of Asturias, Spain (Proyect: FC-15-GRUPIN14-053).

### Conflict of interest statement

The authors declare that the research was conducted in the absence of any commercial or financial relationships that could be construed as a potential conflict of interest.
